# Advance care planning among older adults in Belgium with Turkish backgrounds and palliative care needs: A qualitative interview study

**DOI:** 10.1080/13814788.2023.2271661

**Published:** 2023-10-23

**Authors:** Hakki Demirkapu, Lieve Van den Block, Stéphanie De Maesschalck, Aline De Vleminck, F. Zehra Colak, Dirk Devroey

**Affiliations:** a Department of Family Medicine and Chronic Care, Vrije Universiteit Brussel, Brussels, Belgium; b End-of-Life Care Research Group, Vrije Universiteit Brussel and Ghent University, Brussels and Ghent, Belgium; c Department of Public Health and Primary Health Care, Ghent University, Ghent, Belgium; d Department of Education, University of Utrecht, Utrecht, The Netherlands; e Department of Family Medicine and Chronic Care, Vrije Universiteit Brussel [VUB], Brussels, Belgium

**Keywords:** Advance care planning, end-of-life care, older adult, migrant, qualitative study

## Abstract

**Background:**

Data on advance care planning (ACP) among migrants in Europe is lacking. Research has shown that few older migrants in the United States perform ACP due to healthcare system distrust, collectivistic values and spirituality/religion.

**Objectives:**

To explore the ACP knowledge and perspectives of older Turkish-origin adults in Belgium requiring palliative care.

**Method:**

General practitioners (GPs) in Brussels and Antwerp recruited Turkish-origin participants aged ≥ 65 years with palliative care eligibility for this qualitative study. A GP conducted semi-structured interviews in Turkish in respondents’ homes between May 2019 and February 2022 using a topic guide. Two researchers performed combined inductive/deductive thematic data analysis.

**Results:**

All 15 interviewees (average age, 79 years) lacked ACP awareness and information. Some had discussed specific end-of-life preferences (e.g. care location, burial place) with family. Still, many did not feel the need to discuss future healthcare preferences, due mainly to trust in God and family for caretaking and decision-making. Some respondents viewed ACP discussions as applicable, relieving the burden on family and enabling proactive addressing of ‘what if’ questions. Self-identified ACP barriers were fear of making wrong decisions, ‘living in the moment’ and difficulty discussing death. Facilitators were obtaining sufficient ACP information and recent family illness or death.

**Conclusion:**

Our sample of Turkish-origin older adults in Belgium requiring palliative care lacked ACP knowledge. Our findings suggest that their lack of engagement in discussing end-of-life medical care planning was linked to their family dynamics and religion. The findings have implications for healthcare providers to ethnic-minority groups.

KEY MESSAGES

- The Turkish-origin older adults in Belgium with palliative care needs who participated in this study lacked knowledge of advance care planning.
- This group’s lack of engagement in discussing end-of-life medical care planning may be linked to their family dynamics and religious backgrounds.

## Introduction

Advance care planning (ACP) is defined by European consensus as ‘enabling individuals to define goals and preferences for future medical treatment and care, to discuss these goals and preferences with family and healthcare providers, and to record and review these preferences if appropriate’ [1]. It allows patients to articulate future care priorities and caregivers to best meet their care objectives should physical and/or mental health deterioration inhibit patient communication. ACP initiation is indicated upon worsening health, ageing, palliative care initiation and/or residential care home admission [1].

ACP allows patients to maintain control and peace of mind, increasing their satisfaction and quality of life [2]. It reduces the ambiguity of patient-family communication and families’ decision-making burden and anxiety [2,3]. It increases advance directive creation and reduces care providers’ ethical dilemmas [3]. However, ACP does not consistently achieve these outcomes or improve subsequent care, mainly when it consists only of establishing advanced end-of-life care directives [4]. A recent scoping review of randomised controlled trials revealed inconsistent findings concerning ACP’s effects on care quality, health outcomes and healthcare utilisation [5].

ACP and the designation of legal patient representatives are not prevalent in Belgium and are related primarily to the refusal of life-prolonging treatment [6,7]. In the United States, ACP uptake is lower in certain migrant populations than among older White Americans; across ethnic-minority groups, worse health status and good ACP knowledge are facilitators, and healthcare system distrust is a barrier [8]. Collectivistic cultural values and spirituality/religion influence ACP engagement [8]; the philosophy underlying ACP may be difficult to reconcile with prevailing norms and values in some non-Western cultures, preventing successful implementation [9]. For migrant populations in Europe, high-quality data to substantiate these hypotheses are lacking.

People of Turkish origin form one of the largest non-Western groups in Belgium (comprising 2.1% of the population) and nearby European countries [10]. Ageing Turkish immigrants in Belgium increasingly require formal care but their health system access and receipt of appropriate end-of-life care are hindered by a lack of knowledge about the system, language barriers, low education and health literacy, precarious financial situations and the perceived insensitivity of the system to their cultural and religious attitudes and values [11].

We previously explored the ACP knowledge, attitudes, barriers, facilitators and experiences of older Turkish adults in Belgium with chronic illnesses but no palliative care need [12]. Age and health status affect ACP uptake, and ACP engagement is lower among migrants than non-migrants with serious illnesses in the United States [9,13]. The ACP-related needs and views of patients with progressive, incurable conditions likely differ from those of healthier adults [13]. In this qualitative study, we examined ACP knowledge, experiences, views, facilitators and barriers in the under-researched population of older Turkish-origin patients in Belgium requiring palliative care.

## Method

### Study design

We explored respondents’ knowledge of and perspectives about ACP, especially end-of-life medical care planning, by conducting face-to-face semi-structured interviews in Turkish. We followed the consolidated criteria for reporting qualitative research in describing the methods and findings [14].

### Patient recruitment

The principal researcher (HD) asked general practitioners (GPs) serving older Turkish adults in Brussels and Antwerp, identified *via* primary care and the authors’ networks, to recruit Turkish-origin people aged ≥ 65 years who were living in Belgium and eligible for palliative care, according to the Palliative Care Indicator Tool (PICT) [15]. The GPs identified patients born in Turkey based on their knowledge of their personal histories and medical records. According to the PICT criteria, older people with incurable diseases and two or more frailty indicators were included [15]. Those diagnosed by their GPs with cognitive impairment or dementia were excluded.

The GPs asked eligible individuals whether they were interested in a face-to-face interview about possible future care planning with a researcher/medical doctor in Turkish. The researcher contacted consenting individuals by telephone to provide further information, ascertain their willingness to participate and schedule home interviews with willing participants. Participants were recruited until no new relevant knowledge had been obtained in three previous interviews (i.e. data saturation).

### Data collection

The principal researcher (HD), a male GP fluent in Turkish with experience in qualitative research and semi-structured interviews, conducted all interviews (one per participant; average length, 37 min) between May 2019 and February 2022. Each interviewee provided oral and written consent in Turkish, with the interviewer’s and/or family members’ assistance when needed, and the interviews were then audio recorded with two devices (to avoid information loss in case of device malfunction). The face-to-face approach was adopted to collect in-depth, contextualised information [16]. All 2019 coronavirus disease-related safety measures (i.e. masking, hand disinfection and distancing) were taken. The researcher recorded participants’ socio-demographic characteristics at the ends of the interviews and any additional relevant information thereafter.

Fifteen respondents (10 men, 5 women) aged 65–89 (mean, 79; median, 77) years were interviewed. Three individuals approached by their GPs were not interested in participating. All interviewees were first-generation immigrants to Belgium and identified as Muslim. Respondents’ children were present during 13 interviews, as they lived with their parents and/or were curious. Six respondents mentioned being in poor health. Most had low educational levels; only two respondents had completed high school (Table 1).

**Table 1.** Participants’ socio-demographic characteristics (*n* = 15).

Characteristic	Category	*n*
Age (years)	65–75	6
	80–89	9
Gender	Man	10
	Woman	5
Migration background	First generation	15
Educational level	Unable to read/write	3
	Some primary school (incomplete)	5
	Primary education	5
	High school or college	2
Profession before retirement	Homemaker	3
	Labourer	12
Marital status	Married	8
	Widowed	7
Living situation	Alone	3
	With spouse	5
	With children or spouse and children	7
Residence in Belgium (years)	20–45	3
	>45	12
Number of children	2, 3	4
	4, 5	7
	≥6	4
Serious illness^a^	Cancer	6
	Serious respiratory dysfunction	6
	Cerebrovascular accident	2
	Stage-4/5 renal failure	3
	NYHA class-4 heart failure	4
Self-perceived health	Good	1
	Neutral	8
	Poor	6
GP background	Turkish	10
	Belgian	5
Residence	Rural	9
	Urban	6

^a^ Five respondents had multiple serious illnesses.

NYHA: New York Heart Association; GP: general practitioner.

We used the interview guide developed, forward-backwards translated from English to Turkish, pilot tested and revised for content clarity in our previous study [12]. It contains open-ended questions about interviewees’ ACP-related knowledge, experiences, views, facilitators and barriers (Supplementary Box 1). The interviewer first asked about participants’ ACP knowledge without explaining the concept. He then introduced ACP as a form of advanced communication about the care a person would (not) like to receive should they no longer be able to communicate such preferences [1]. While assessing participants’ ACP views, he explained the concept in greater detail. The interviews focused on aspects of end-of-life medical care planning that conveyed ACP-related themes, such as preferences about life-prolonging treatment, care location (e.g. moving to a residential home) and power of attorney, with examples. The interviewer also described an example case to explain ACP use in lay terms.

### Data processing and analysis

The interviews were transcribed verbatim. Two researchers (HD and FZC) independently conducted a combined deductive/inductive thematic analysis of all transcripts [17]. The transcripts were read line by line, and the data were assigned to five a-priori-defined categories, based on the topic guide (deductive component; Supplementary Box 2). Data in each category were further coded into subcategories (inductive component), grouped to form themes. Code list recording and analysis were performed using the NVivo12 software (QSR International, Melbourne, Australia). The researchers, fluent in Turkish and English, worked independently to develop findings without meaning loss, enhance transparency during translation and manage sensitive data. They first analysed the Turkish transcripts, then forward-backward translated relevant portions into English and repeated the analysis with the English transcripts. When direct translation into English did not yield a clear result, indirect translation with meaning retention was performed (e.g. ‘don’t connect me to a ventilator’ instead of ‘don’t connect me to the machine’). The bilingual analysis permitted the consideration of cultural context and meaning-based interpretation and translation to most accurately reflect participants’ experiences [18]. Data collection and analysis were conducted concurrently with the interviews to pursue emerging lines of inquiry. The research team held monthly discussions to enhance triangulation, limit bias and ensure the reliability of interpretations.

## Results

In three cases, respondents’ children asked the interviewer beforehand (*via* telephone or in-person with the respondents absent) to avoid mentioning ‘bad’ diagnoses or prognoses, which they explicitly stated were undisclosed. The interviewer respected these requests. One son cautioned:

*‘Don’t mention her [his mother’s] illness [lung cancer] before her! The doctor revealed her diagnosis but we told her it was COVID-19-related and would pass. We managed to convince her that she has no significant illness right now.’ (son of Respondent 15)*

The ACP-related themes identified were the lack of ACP knowledge but some end-of-life care discussions with family, trust in God and family and positive ACP views. The barriers identified were the fear of making wrong decisions, ‘living in the moment’ and the fear of death. Receiving sufficient ACP information and recent family illness or death were identified as facilitators.

### Lack of ACP knowledge but some end-of-life care discussions with family

Before the interview, no respondent had heard the term ‘advance care planning’. However, some respondents had discussed their preferences for future care should they develop serious illnesses or otherwise be in deteriorating health with family members:

*‘I keep praying to God, ‘please don’t let me be bedridden’. Bedridden people are so miserable. Who would help me eat or go to the toilet? I was talking to [grandchild] and said, ‘you can’t take care of me once I’m bedridden. If that happens, leave me in a hospital or hire someone to care for me at home.’ (Respondent 12, 86-year-old woman)*

Others had discussed their preferred care and burial locations with family members because they were aware of the life-limiting nature of their illnesses:

*‘[My] illness is severe. The chemotherapy is tough on me … Of course I’ve talked to my family already about where I’d like to be buried. I told them, ‘just don’t bury me here [in Belgium].’ (Respondent 10, 65-year-old man)*

Only one respondent had informed his family of his wish not to receive life-prolonging treatment should his condition worsen:

*‘I told my family to let me go without any fuss. I don’t want my life prolonged. What else is there to talk about? I am content with what God has given me. Death is a difficult thing [but] if you’re connected to [life-prolonging] machines, you’ll only suffer.’ (Respondent 5, 80-year-old man)*

### Trust in God and family

Most respondents considered end-of-life care discussions futile, repeatedly mentioning their faith in God (as the only one who knows/decides what the future holds) and confidence in their relatives. They wished only to die with faith and were not interested in end of life-related medical discussions.

*‘I don’t want to talk about [life-prolonging treatment]. I don’t want to decide, life and death are in God’s hands. I wouldn’t take that responsibility away from Him.’ (Respondent 2, 83-year-old man)*

They also trusted that their relatives (mainly spouses and children) would take charge and know what to do should their health deteriorate beyond decision-making capacity, obviating the need for a power of attorney or discussions about life-prolonging treatments.

Some participants who trusted in God and family, but required more care, had discussed their preferences (e.g. for care location) with family members:

*‘God is great. No worries with God… I leave these things [end-of-life medical care] to my children. They decide … I said to my children that I want to go to a nursing home. I don’t want to burden them anymore. ‘(Respondent 15, 71-year-old woman)*

Some family members interrupted interviews to volunteer their views on ACP. They emphasised that their parents were looked after well and that this was sufficient, suggesting that we interview seriously ill people, whom they considered to be ready for such discussions:

*‘She [his mother] is good now. Her condition is improving, and the wound is healing. So there’s no need to talk about [end-of-life] matters. We [Turks] don’t have particular preferences; everything comes from God. You should instead ask these questions of those nearing death. If, God forbid, she [his mother] was bedridden today, then she might want to [discuss ACP]. But for now, she feels in good health’. (son of Respondent 15)*

Some children indicated that they intuitively understood their parents’ wishes and no discussion was required:

*‘My father’s wishes are clear to us. We haven’t had discussions on this topic [ACP], but we know his wishes … we have that confidence [between parent and child] … There’s no need, we’ll take good care of him … everything is clear, the plan is clear – he will stay here [at home].’ (son of Respondent 14)*

### Positive views on ACP

After ACP was explained with examples, some respondents considered the discussion of end-of-life care preferences with relatives and physicians (primarily GPs) valid and wanted to involve close family members. They mentioned the confidence that would come from having made plans in advance of deteriorating physical or mental health:

*‘It’s good to talk in advance as it gives them [children] the chance to ask, ‘tell us what you want before you die’. It’s a good thing. Talking openly gives confidence; it’s suitable for everyone.’ (Respondent 1, 80-year-old woman)*

They viewed the discussion of topics such as life-prolonging treatments, care location and power of attorney as increasing certainty:

*‘It’s beneficial to say it all beforehand when your eyes are still open [i.e. conscious], explaining, for example, ‘I want this but not that [treatment]’. There’s no need to hide these [preferences] from family.’ (Respondent 4, 75-year-old man)*

They felt that advanced specification of their preferences could prevent their suffering and avoid unduly burdening their families should the need for intensive care arise:

*‘This [ACP] should be discussed while you are healthy and conscious. I’m healthy now, but God only knows what will happen tomorrow. I don’t want to burden my children. I can tell them, ‘I don’t want to go to a nursing home’. Also, I don’t want to be miserable at the end. I will tell my doctor [GP], ‘If I’m really ailing and can’t talk, don’t connect me to a ventilator’. When we talk about it [beforehand], then my son can say, ‘my mother’s wishes are this’. Or my doctor. He would be able to say, ‘she told me [her end-of-life care preferences]’. (Respondent 9, 70-year-old woman)*

### ACP barriers

#### Fear of making wrong decisions

After explaining ACP and providing examples, some respondents emphasised their lack of education and fear of making bad decisions or answering their doctors incorrectly. They felt uncertain about their ability to discuss end-of-life medical care, preferring to avoid discussions about life-prolonging treatments and leaving such decisions to their adult children:

*‘Talking beforehand is very good, but I don’t understand much. I didn’t even finish primary school and I’m afraid of making the wrong decision. I don’t know anything, but he [son] does. My children can decide.’ (Respondent 6, 87-year-old man)*

##### ‘Living in the moment’ attitude

Some respondents were convinced that their current conditions were not critical, death was not imminent and it was better to take things as they come:

*‘My current situation is okay, although I can’t clean and get to the shops like before. I don’t think about the future because my health is not too bad right now. But if later I can’t walk, then it will be different. ‘(Respondent 12, 86-year-old woman)*

Some expressed anxiety in this context:

*‘I get anxious about what will happen to me. But I remind myself that God is great, and where there is God, there is no worry. After that, I feel much better. What should I say? I’ll talk when things worsen; there’s no need right now.’ (Respondent 15, 71-year-old woman)*

### Difficulty talking about death

A few respondents were reluctant to discuss end-of-life care preferences because of the psychological discomfort of being reminded of death:

*‘I haven’t thought about it [end-of-life care] until today. No one wants to think about his death; it’s too difficult to talk about.’ (Respondent 7, 70-year-old man)*

### ACP facilitators

#### Obtaining ACP information

The most frequently mentioned ACP facilitator was obtaining ACP information. With sufficient information and opportunities to ask questions, respondents indicated they would feel comfortable expressing their wishes to their GPs and delegating a power of attorney.

*‘If we know enough about [ACP], we could tell them [children], ‘the doctor informed us about [ACP], and now we should discuss it together’. I could say, ‘If I get worse, you are authorised to make decisions; I grant you this authority’. (Respondent 13, 73-year-old man)*

##### Family experience of severe illness or death

Some respondents’ recent family experiences of severe illness or death prompted them to think and talk with relatives about their end-of-life care:

*‘My mother died within 40 days of my son-in-law. After all this bad news, I started thinking about it [ACP]. We all die, but after [a recent fall], I’m giving it more thought. I don’t want to go into care; I want to die at home. I’ll share my preferences with my children when I go to Turkey this year.’ (Respondent 9, 70-year-old woman)*

## Discussion

### Main findings

The older Turkish-origin interviewees requiring palliative care in Belgium lacked ACP awareness and detailed information. Some had discussed their end-of-life preferences with family, but most felt no need to do so, due mainly to their trust in God and family for caretaking and decision-making. Some respondents viewed such discussions as beneficial, mainly because they would relieve the burden on families and proactively address ‘what if’ questions. ACP barriers were the fear of making wrong decisions, ‘living in the moment’ and difficulty discussing death. Facilitators were the receipt of sufficient ACP information and recent family illness or death.

### Strengths and limitations

This study’s strengths include the interviewer’s knowledge of the participant’s native language and cultural background, which enabled direct, nuanced conversation. The respondents’ socio-demographic characteristics align with those of the general population of older Turkish-origin adults in Belgium [19], supporting the transferability of our findings.

This research also has limitations. Interviewees’ responses may have been biased due to family members’ presence, and sometimes unprompted participation in most interviews. However, we allowed family members to be present in cultural alignment with their essential care roles, including end-of-life decision-making [11,20]. Most interviewees’ unawareness of the seriousness of their health conditions may have influenced their views and willingness to engage in ACP. The interviewer’s authority-figure position as a male medical doctor may have influenced the respondents’ assertiveness in their responses. Additionally, we focused on people with Turkish backgrounds in Belgium who were eligible for palliative care; the findings may be relevant for other groups with similar immigration histories, as found for Moroccans in Belgium without palliative care needs [21]. However, additional research is needed to understand the experiences of migrants from other ethnic groups requiring palliative care in other nations.

### Comparison with existing literature

Our participants’ lack of ACP knowledge is consistent with findings for other cultural-minority groups, underlain by low educational levels, language barriers, and the lack of tailored information [9,22]. Their age, education, chronic conditions and the language barrier likely contributed to their low health literacy, which is common and affects the likelihood of pursuing ACP among migrants [8,9]. Health literacy impacts patients’ ability to understand and make decisions about their health and healthcare [23]. Our respondents were deemed by GPs to require palliative care and to be likely to die within 6–12 months, but they did not view death as imminent or discussions about end-of-life care preferences as necessary.

The ACP views of older Turkish adults in Belgium differ according to the palliative care need. In contrast to the present findings, most of those without such need considered ACP discussions useful and ready to engage [12]. Family members are often increasingly current for medical discussions as patients’ care needs increase, possibly increasing their reticence regarding end-of-life care preferences. However, some interviewees in this study who required more care and/or recognised the severity of their conditions had talked with family about their preferred care and burial locations. Previous research confirmed that Turkish-origin older adults in Belgium prepare for their funerals, desiring to be buried ‘at home’ and according to Islamic custom in Turkey [24]. Our respondents did not mention language issues as an ACP barrier, perhaps because the interviews were language-concordant, although older Turkish adults mentioned the language barrier in our previous language-concordant research [12]. Another possible reason is the Turkish origin of most participants’ GPs, which was not the case in our earlier study.

The significant influence of the respondents’ values on ACP engagement is consistent with findings for people with migration backgrounds [9]. Older first-generation Turkish migrants in Belgium have been characterised as traditional, collectivist and family-centred, with close family members primarily responsible for (end-of-life) care decisions [11,20]. Most respondents expressed confidence that their children would care for them according to traditional filial responsibility and in line with some relatives’ insistence during interviews that the family had the situation under control, seemingly discomfited at the suggestion that their parents might go to live in a care home. Other respondents wished to involve family members in ACP conversations, citing the value of avoiding worry or encumbrance. These and previous findings demonstrate that end-of-life decisions are affected by others’ concerns and opinions [25,26].

Given their faith, many respondents expressed few worries about the future and felt no need to discuss end-of-life care preferences. They viewed God as determinative of all physical and spiritual well-being (including life and death) [9,22]. Individuals tend to become more religious with age to relieve illness-, loss- and death-associated stress [27]. Most respondents in our previous study did not cite religion as an ACP barrier, perhaps because they were younger on average [12]. Minority-group members’ completion of ACP documents declines with increasing religiosity and positive spiritual coping [9, 22]. We also assume that our participants’ religious expressions reflected their reluctance to discuss the topic further.

This study’s ACP examples and cases enabled deeper discussion, beyond concrete ACP aspects. We obtained meaningful insights into our respondents’ thoughts, feelings, religious beliefs and cultural values, consistent with a recent public-health palliative care ACP approach that emphasises individuals’ priorities, values and lived experiences [28]. ACP discussions should focus on what matters most to people rather than narrowly emphasising harm reduction (i.e. avoiding unwanted treatments). Such approaches enable tailored planning and goal-oriented end-of-life care underpinned by patients’ contexts and values, improving their experiences [29,30].

The ACP barriers and facilitators cited by our respondents were similar to those of native-Belgian older adults with limited prognoses (fearing death, trusting in God and family and non-acknowledgement of the end of life as barriers and bad experiences with death and loved ones’ deaths as facilitators) in a qualitative study [30]. A marked difference was that many older native-Belgian adults were willing to discuss death and plan end-of-life care; some had written advance directives and/or ensured that their preferences were specified in their medical records [30]. Similar to our respondents, they were less interested in planning for end-of-life care aspects such as life-sustaining treatments [30]. In contrast to our respondents, native Belgians cited their limited trust in surrogates and wish to maintain control over end-of-life care as ACP facilitators. This difference could reflect our respondents’ profound trust in family and fear of making wrong decisions.

### Implications for practice and research

Providers of end-of-life care to members of collectivistic cultures (e.g. Turkish) should recognise the salience of family and, with patients’ permission, involve close relatives in ACP discussions. The decision to involve family does not necessarily impede ACP discussions, as it can be seen as an aspect of patient autonomy [26]. Research conducted with family caregivers could provide more insight into their views, allowing healthcare providers to adapt their approaches to ACP discussion with Turkish-origin patients. Current ACP recommendations [1] highlight the importance of starting ACP conversations even without a life-threatening diagnosis. Healthcare providers could use ACP examples to determine their patients’ views and wishes. The connection between diagnostic/prognostic disclosure and ACP could be investigated further from patients’, family members’ and healthcare providers’ perspectives.

## Conclusion

Our sample of Turkish-origin older adults in Belgium requiring palliative care lacked ACP knowledge. The findings suggest their lack of engagement in discussing end-of-life care planning is linked to their family dynamics and religion. They have implications for healthcare providers to ethnic-minority groups.

## Ethical approval

The Medical Ethics Commission of Brussels University Hospital approved this study (B.U.N. 143201838280), registered at ClinicalTrials.gov (no. NCT03930823). All data were pseudonymised.

## Research ethics and patient consent

The study design was approved by the medical ethics committee of Brussels University Hospital (B.U.N. 143201838280, 6 February 2019) and registered at ClinicalTrials.gov (no. NCT03930823). The interviews were conducted after receiving written and verbal consent, including for the publication of anonymised findings, from the participants.

## Supplementary Material

Supplemental Material

Supplemental Material

## Data Availability

All requests for data access should be addressed to the Chief Investigator at hakki.demirkapu@vub.be and will be reviewed by all authors.
