# Supplementary material for: Advance care planning among older adults in Belgium with Turkish backgrounds and palliative care needs: A qualitative interview study
Source: Eur J Gen Pract. 2023 Oct 23;29(1):2271661. doi: 10.1080/13814788.2023.2271661 (PMC10990253; doi:10.1080/13814788.2023.2271661)
Supplement: Supplemental Material [file IGEN_A_2271661_SM3448.docx]

**Supplementary Box 2.** Categories used for data coding.

| 1. Knowledge about ACP 2. Experience with ACP 3. Views about ACP 4. Barriers to ACP 5. Facilitators of ACP |
| --- |

ACP, advance care planning.
