# Supplementary material for: Advance care planning among older adults in Belgium with Turkish backgrounds and palliative care needs: A qualitative interview study
Source: Eur J Gen Pract. 2023 Oct 23;29(1):2271661. doi: 10.1080/13814788.2023.2271661 (PMC10990253; doi:10.1080/13814788.2023.2271661)
Supplement: Supplemental Material [file IGEN_A_2271661_SM3447.docx]

**Supplementary Box 1.** Interview topic guide.

| **Introductory questions**  How would you describe your state of health?  Do you have any questions regarding your concerns and worries about your healthcare in the latter part of your life?  Have you spoken to anyone about these matters? Why/why not? Have you done something about your concerns and worries? Have you planned something for the future?  Can you explain further?  How do you feel about this?   \| **What knowledge does the respondent have about advance care planning?**  What do you know about advance care planning? (If the interviewee knows nothing, explain):  Advance care planning is thinking ahead about the future. This is about discussing your future healthcare desires with your relatives and doctor. It is about voicing your wishes while you are still in good health and can express your thoughts and wishes. Older people do this planning to communicate their thoughts and wishes regarding future healthcare and even end-of-life situations. Such planning is essential for people to prepare for situations where they cannot speak or make decisions due to their future illnesses. Thus, it enables others to consider your desires as much as possible. Did you know you could communicate your wishes in advance for such situations? \| \| --- \| \| **What experience does the respondent have?**  Some older people might have already experienced instances when this advanced healthcare planning would have been helpful with some of their relatives. For example, suppose a relative falls seriously ill or has an accident and loses consciousness. In that case, it is up to the family and the doctor to make decisions on their behalf because of the patient’s incapacity to communicate their own wishes. Have you come across something like this?  Have you ever thought about your future healthcare? \| |
| --- | --- | --- |
| \| **What are the respondents’ views about advance care planning?**  Before you become severely ill, do you wish to discuss your future wishes with someone in advance? Why/why not? How do you feel about discussing illness and death?  For some people, their wishes must be known by others to prepare themselves for future situations where they cannot express their wishes and make decisions because their physical or mental condition is inferior. I am now going to give you three examples of these subjects.  1. It is now possible to slightly prolong the life of someone seriously ill, is not conscious, and has no hope of recovery by drip feeding them, on a ventilator, and using antibiotics. While you are in good health, you can refuse such treatments, should you ever fall into that state. What do you think about discussing this now for the future?  2. You can now specify that you would like to move into a residential home when you are very old and unable to look after yourself anymore. What do you think about discussing this now for the future?  3. You can give someone authority (power of attorney) to act on your behalf. Suppose at some stage you fall seriously ill and can’t think properly or make decisions about your treatment. In that case, this person will be able to make decisions for you and ensure that your previously communicated wishes are fulfilled.  What do you think about discussing this now for the future? Do you wish to give authority on your behalf to someone? Why/why not?  Even if you do not have such a severe illness now, what are the advantages and disadvantages of discussing your wishes for the future?  I will now give you an example of an advanced care plan. A 75-year-old patient has Alzheimer’s (memory loss) disease. She lives with her daughter. She says to her doctor, ‘if I ever have a serious illness from which I will not recover, I do not wish to be wired up to machines in the hospital, like my husband. He was in pain for weeks before he died.’ So, one day this patient falls ill. She has a high temperature, is coughing, and cannot eat or drink. Her general practitioner wants to hospitalise her as she may recover through intravenous (through a drip) feeding and antibiotics. The family doctor and her daughter decide that if she does not improve within a few days, they will stop the treatments and discharge her to spend her last days at home. The patient recovers after three days and returns home. Three years later, her dementia has become much worse. The patient can no longer get out of bed or her armchair and has stopped eating. Nothing gives her pleasure anymore. She does not even recognise her daughter. She is not able to make decisions about her treatment. Her daughter says, ‘how about feeding her with a drip?’ Her doctor says that he does not wish to subject the patient to treatment anymore because when she was healthy, she had expressed her wish not to be connected to any machines if she ever contracted such a severe illness. After two painless months, the patient dies. What do you think about this story?  What do you think about the patient communicating her wishes in advance while she is still healthy/conscious? \| \| --- \| \| **What are the barriers and facilitators?**  In your opinion, what makes it easier or what would make it easier to talk about these subjects?  In your opinion, what makes it harder to talk about these subjects? \| |
